# CSF Free Light Chains as a Marker of Intrathecal Immunoglobulin Synthesis in Multiple Sclerosis: A Blood-CSF Barrier Related Evaluation in a Large Cohort

**DOI:** 10.3389/fimmu.2019.00641

**Published:** 2019-03-29

**Authors:** Makbule Senel, Fatemeh Mojib-Yezdani, Ulrike Braisch, Franziska Bachhuber, Jan Lewerenz, Albert C. Ludolph, Markus Otto, Hayrettin Tumani

**Affiliations:** ^1^Department of Neurology, University of Ulm, Ulm, Germany; ^2^Institute of Epidemiology and Medical Biometry, Ulm University, Ulm, Germany; ^3^Specialty Hospital of Neurology Dietenbronn, Schwendi, Germany

**Keywords:** immunoglobulin free light chains, oligoclonal bands, OCB, intrathecal IgG synthesis, IgG index, multiple sclerosis, cerebrospinal fluid, serum

## Abstract

**Objectives:** The importance of immunoglobulin G (IgG) oligoclonal bands (OCB) in the diagnosis of multiple sclerosis (MS) was reaffirmed again in the recently revised MS diagnostic criteria. Since OCB testing is based on non-quantitative techniques and demands considerable methodological experience, measurement of CSF immunoglobulin free light chains (FLC) has been suggested as quantitative alternative to OCB. We aimed to establish reference values for FLC measures and evaluate their diagnostic accuracy with regard to the diagnosis of MS.

**Methods:** Immunoglobulin kappa (KFLC) and lambda (LFLC) free light chains were prospectively measured by nephelometry in CSF and serum sample pairs in 1,224 patients. The analyzed cohort included patients with MS, other autoimmune or infectious inflammatory diseases of the nervous system as well as 989 patients without signs for nervous system inflammation.

**Results:** Regarding diagnosis of MS, the diagnostic sensitivity and specificity of intrathecal KFLC ratio were 93.3 and 93.7% using the CSF-serum albumin ratio-dependent reference values, 92.0 and 95.9% for intrathecal KFLC ratio applying the ROC-curve determined cut-off levels, 62.7 and 98.3% for IgG index, 64.0 and 98.8% for intrathecal IgG synthesis according to Reiber diagrams, and 94.7 and 93.3% for OCB. Diagnostic sensitivity and specificity of intrathecal LFLC were clearly lower than KFLC.

**Conclusions:** Intrathecal KFLC and OCB showed the highest diagnostic sensitivities for MS. However, specificity was slightly lower compared to other quantitative IgG parameters. Consequently, CSF FLC may not replace OCB, but it may support diagnosis in MS as a quantitative parameter.

## Introduction

Multiple sclerosis (MS) is a chronic inflammatory demyelinating disease of the central nervous system (CNS) affecting predominantly young adults and leading to neurological disability (1–3). CSF investigation is indispensable in the diagnostic process of MS and the detection of immunoglobulin G (IgG) oligoclonal bands (OCB) again gained more importance in the recently revised MS diagnostic criteria (4). So far, OCB are the most widely used CSF test to support or rule out the diagnosis of MS (5–7). Furthermore, OCB offer prognostic information concerning the development of MS after a first clinical suggestive event, known as clinically isolated syndrome (CIS) (8, 9). In these cases, detection of OCB has prognostic relevance and can help to identify patients with a high risk of future relapses. However, determination of OCB using isoelectric focusing (IEF) on gels followed by immunoblotting demands considerable methodological expertise and is both labor-intensive and difficult to standardize (10). Human IgG molecules contain two identical heavy chains and two identical light chains, which exist either as kappa or lambda isotypes and are linked to the heavy chains by covalent and non-covalent bonds (11, 12). During the production of intact immunoglobulins, B cells produce an excess of kappa and lambda light chains, which are secreted as free light chains (FLC) (i.e., not bound to heavy chains within an Ig) (13). These FLC can exist as monomers (22–27 kDa, usually kappa) or dimers (44–55 kDa, usually lambda) (14), and can be detected in many biological fluids including serum, urine, synovial fluid as well as, in the CSF (15, 16).

Several studies have indicated that elevated immunoglobulin kappa (KFLC) and lambda (LFLC) free light chains in the CSF may represent a quantitative tool to demonstrate intrathecal IgG synthesis and thereby support the diagnosis of MS (17–28), some even proposing FLC quantification as an alternative to OCB analysis (29, 30). However, diverse methods, both qualitative, e.g., IEF with immunoblotting (31), and quantitative, e.g., radioimmunoassay (24), ELISA (18), and nephelometry (20–22), have been applied for FLC determination. In addition, divergent approaches to calculate intrathecal FLC synthesis were employed, e.g., FLC CSF-serum ratios, CSF KFLC to LFLC ratio, and FLC index. In summary, comparability between the published studies is limited due to different methodologies, lack of appropriate disease controls (usually non-inflammatory neurological cases were used as controls with a lack of other autoimmune CNS diseases than MS), and finally rarity of prospective data.

The aim of the present study was (i) to prospectively measure FLC (both KFLC and LFLC) in CSF and serum by nephelometry in a large cohort, (ii) to establish reference values for FLC as a function of the blood-CSF barrier function based on patients without any clinical and laboratory signs for nervous system inflammation, and (iii) to compare the diagnostic value of different previously proposed methods to calculate intrathecal FLC synthesis, e.g., CSF-serum ratio of FLC (Q FLC), FLC index (Q FLC/Q Albumin), CSF KFLC-LFLC ratio, with well-proven indicators of intrathecal IgG synthesis (OCB and IgG Index) within the same cohort.

## Methods

### Patients

Cross-sectional data of CSF and serum sample pairs of 1,224 patients from the Department of Neurology, University of Ulm (Germany) were investigated prospectively over a period of 18 months.

The analyzed cohort included 75 patients with multiple sclerosis (MS), diagnosed according to the modified McDonald criteria (32), five with clinically isolated syndrome (CIS), 36 patients with other autoimmune CNS diseases (AI-CNS-D), 13 with chronic inflammatory demyelinating polyneuropathy (CIDP), 13 with Guillain-Barré syndrome (GBS), 29 with viral and bacterial CNS infection (CNS-I), seven with CNS tumor, 38 with post-infectious CSF syndrome (P-CNS-I), five with metabolic encephalopathy (ME), 14 with paraproteinemic neuropathy and/or neuropathy with monoclonal gammopathy of unknown significance (PP-PNP), and 989 patients without any signs of nervous system inflammation (no signs of inflammation in CSF, no clinical signs of inflammation, no signs of blood contamination in CSF, and no evidence of haemorrhagic or inflammatory lesion in cerebral MRI, NIND) (Table 1). Lumbar puncture was performed as part of the routine diagnostic work-up. All samples were handled and stored in accordance with BioMS guidelines (33).

**Table 1:** Demographic data and basic cerebrospinal fluid findings.

	***N* (female/male)**	**Age (years)**	**CSF cell count (/μl)**	**Q Alb (× 10^**−3**^)**	**CSF lactate (mmol/L)**	**OCB pos (%)**
MS	75 (56/19)	37 (25–49)	4 (1–10)	5.3 (4.2–6.3)	1.6 (1.5–1.8)	71 (94.7)
AI-CNS-D	36 (18/18)	58 (40–70)	3 (1–5)	7.25 (4.9–10.5)	1.9 (1.6–2.2)	22 (61.1)
CIS	5 (2/3)	31 (28–33)	2 (2–3)	4.7 (3.6–5.0)	1.7 (1.5–1.8)	5 (100.0)
CIDP	13 (5/9)	66 (60–75)	1 (1–1)	10.6 (8.9–12.6)	1.8 (1.6–2.0)	1 (7.7)
GBS	13 (7/6)	62 (52–75)	2 (1–2)	16.5 (9.8–30.6)	2.1 (1.9–2.4)	0 (0.0)
CNS-I	29 (12/17)	55 (42–74)	52 (13–196)	11.7 (6.2–15.3)	1.9 (1.7–2.6)	12 (41.4)
CNS tumor	7(2/5)	56 (43–76)	1 (1–2)	5.7 (5.5–9.5)	1.9 (1.7–2.3)	1 (14.3)
P-CNS-I	38 (23/15)	51 (36–73)	1 (0–2)	5.2 (3.9–7.6)	1.7 (1.5–2.0)	34 (89.5)
ME	5(3/2)	66 (63–72)	1 (1–3)	10.3 (8.5–11.0)	1.9 (1.9–2.0)	1 (20.0)
PP-PNP	14 (9/5)	71 (53–75)	2 (0–3)	6.6 (5.2–9.1)	1.6 (1.4–1.7)	1 (7.1)
NIND	989 (493/496)	58 (43–72)	1 (0–1)	5.9 (4.5–7.9)	1.7 (1.5–1.9)	0 (0.0)

*Data are shown as numbers or median with interquartile range. AI-CNS-D, other autoimmune disease of the central nervous system; CIDP, chronic inflammatory demyelinating polyneuropathy; CIS, clinically isolated syndrome; CNS, central nervous system; CNS-I, viral and bacterial CNS infection; CSF, cerebrospinal fluid; GBS, Guillain-Barré syndrome; ME, metabolic encephalopathy; MS, multiple sclerosis; PP-PNP, paraproteinemic neuropathy and/or neuropathy with monoclonal gammopathy of unknown significance; NIND, non-inflammatory neurological diseases; OCB, oligoclonal bands; P-CNS-I, post-infectious CSF syndrome*.

### Determination of OCB

OCB were detected by isoelectric focusing (IEF) on polyacrylamide gels followed by immunoblotting using an IgG-specific antibody staining. Paired CSF and serum samples were adjusted for IgG concentrations and analyzed on the same gel run as we previously described in detail (34). OCB were evaluated by at least two long-standing experienced technicians and at least two board-certified neurologists with extensive experience in the field of CSF analysis. Two or more IgG bands restricted to the CSF were rated as positive OCBs.

### Determination of KFLC and LFLC

Immunoglobulin kappa and lambda free light chains (KFLC and LFLC) were measured by nephelometry (Siemens N Latex FLC kappa and lambda assays on Siemens BN ProSpec®) according to the instructions supplied by the manufacturer. The detection antibodies in these assays are monoclonal. All samples were analyzed within the same working day together with other CSF measurements according to the sampling protocol of BioMS (33).

We calculated the CSF-serum ratio of albumin (Q Alb), KFLC (Q KFLC), and LFLC (Q LFLC) and determined the KFLC index (Q KFLC/Q Alb) and LFLC index (Q KFLC/Q Alb) by correcting for Q Alb. In addition, the CSF KFLC to LFLC ratio (CSF KFLC/CSF LFLC) was calculated.

CSF leukocyte count (cells/μl), CSF total protein (g/L), CSF lactate (mmol/L), the albumin CSF-serum concentration ratio (Q Alb), CSF and serum immunoglobulin G, A, and M levels were obtained as previously described (35, 36).

### MRI Analysis

MRI scans of the brain and spinal cord were performed on a 1.5 tesla whole-body MRI (Symphony Siemens, Erlangen, Germany) according to a previously fixed protocol including T1-weighted spin-echo (SE) axial slices with and without application of gadolinium-DTPA as well as T2-weighted SE axial slices.

### Statistical Analysis

Statistical analysis and the graphical representation of the data was performed using SPSS (version 24.0; SPSS Inc., Chicago, IL, USA), GraphPad Prism (version 6.0, GraphPad Software, San Diego, CA, USA), and R software (version 3.4.0). Differences of FLC levels between two disease groups were analyzed using the non-parametric Mann-Whitney U-test due to skewed data. The correlation between two parameters was analyzed using Spearman rank order correlation. *P*-values below 0.05 were considered to be significant.

FLC reference values dependent on Q Alb were estimated by linear quantile regression (37, 38) using the R package quantreg and plotted on the log scale. Receiver Operating Characteristic (ROC) curve analysis, calculating the area under the ROC curve (AUROCC), was used to determine the diagnostic accuracy of FLC values as diagnosis markers for MS. The Youden index (sensitivity+specificity-1) was calculated for a range of cut-off values to find for each FLC value the optimal value with the highest discriminatory accuracy (39).

Sensitivity was calculated as [true-positive/[true-positive + false-negative]], specificity was calculated as [true-negative/[true-negative + false-positive]]. The positive predictive value (PPV) was calculated as [true-positive/[true-positive + false-positive]], and the negative predictive value (NPV) as [true-negative/[true-negative + false-negative]]. For all diagnostic values the exact 95% confidence intervals were given (40).

## Results

### Clinical Findings and Main CSF Examination Results

A total of 1,224 CSF-serum pairs from 1,224 patients were included in this study. Demographic, clinical and main CSF characteristics are summarized in Table 1.

### FLC in Non-inflammatory Neurological Diseases—Establishment of Reference Values

**Figure 1:**
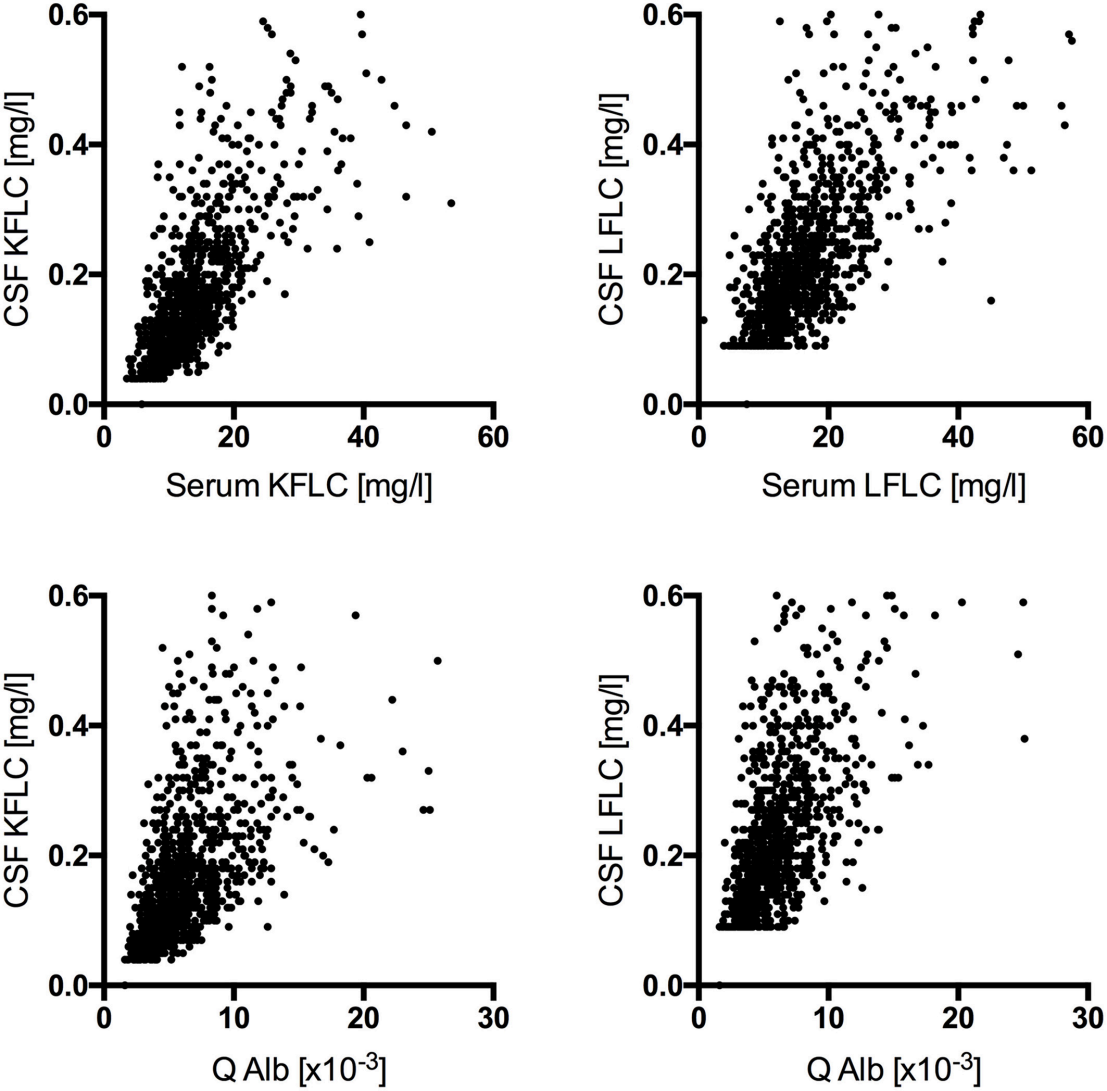
Correlation among cerebrospinal fluid free light chain levels in non-inflammatory neurologic diseases (*n* = 989) with the respective serum levels (KFLC: *p* < 0.001, *r* = 0.744; LFLC: *p* < 0.001, *r* = 0.737) and the respective CSF-serum ratio of albumin (Q Alb) (KFLC: *p* < 0.001, *r* = 0.638; LFLC: *p* < 0.001, *r* = 0.653) are shown.

In NIND (*n* = 989), CSF FLC levels correlated significantly with serum FLC levels (KFLC: *p* < 0.001, *r* = 0.744; LFLC: *p* < 0.001, *r* = 0.737) as well as with the respective Q Alb (KFLC: *p* < 0.001, *r* = 0.638; LFLC: *p* < 0.001, *r* = 0.653) (Figure 1).

**Figure 2:**
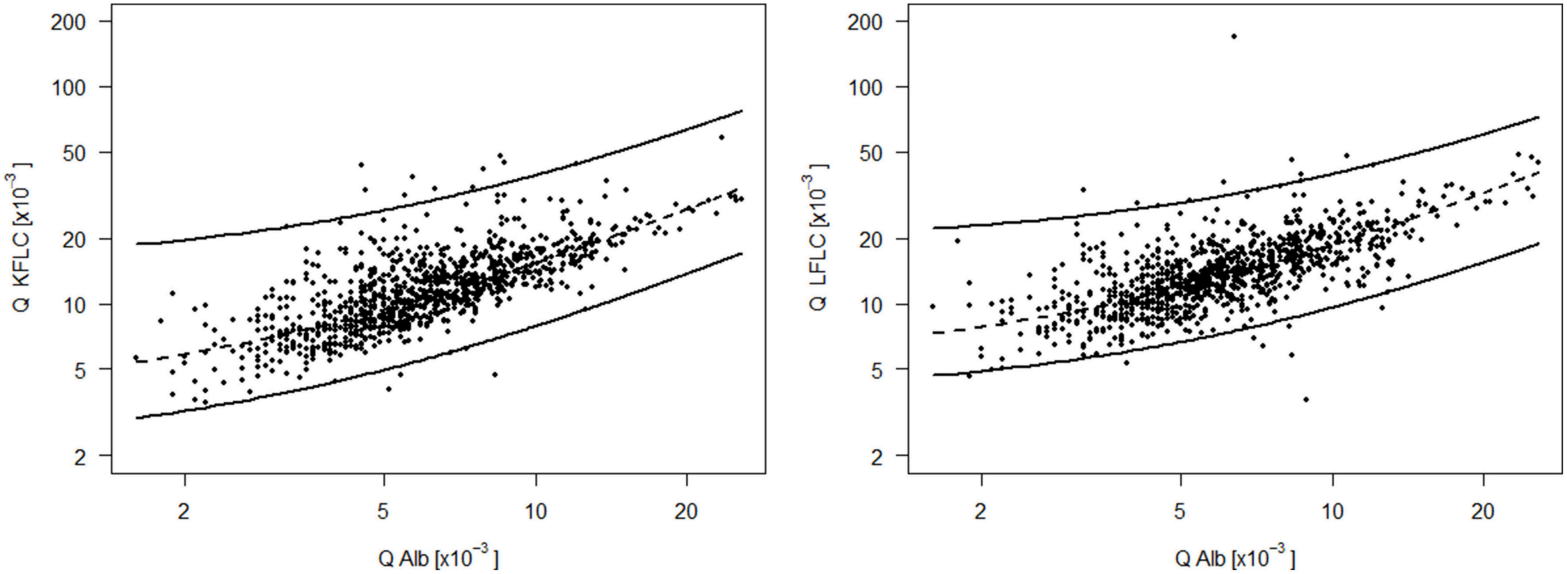
CSF-serum ratio of kappa (Q KFLC) and lambda (Q LFLC) free light chain is plotted against the respective CSF-serum ratio of albumin (Q Alb) on the log scale. The upper line is the 99% quantile estimated by linear quantile regression and indicates the upper reference value of Q KFLC and Q LFLC dependent on Q Alb and based on a control group of 989 non-inflammatory neurologic diseases and a range of Q Alb from 1.6*10^−3^ to 25.7*10^−3^. Formula for the Q Alb dependent upper reference value (upper line): Q KFLC = 9.50 + 2.08*Q Alb; Q LFLC = 16.37 + 1.36*Q Alb. The lower line is the 1% quantile line and the dashed line in the middle is the 50% quantile (median) line.

To establish blood-CSF barrier related reference values for Q FLC, the Q FLC of all NIND was plotted against the respective Q Alb on the log scale. The 99% quantile estimated by linear quantile regression was indicated as the upper reference value of the respective Q FLC (Figure 2). This resulted in the following equations for the Q Alb-dependent upper reference values:

$$\begin{array}{l} \text{QKFLC}=14.85+2.41*\text{QAlb};\text{QLFLC}=18.86+2.06*\text{QAlb}. \end{array}$$

### FLC in CSF and Serum—Group Differences

**Figure 3:**
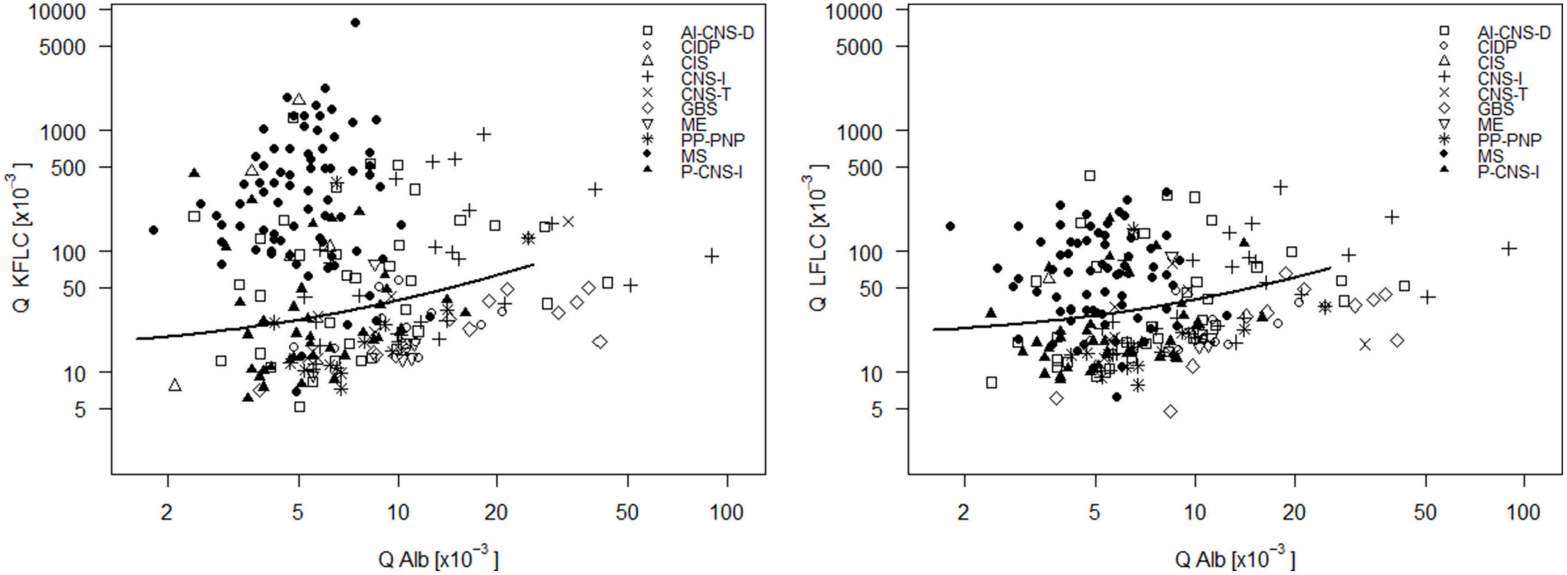
CSF- serum ratio of kappa free light chain (Q KFLC) **(left)** and lambda free light chain (Q LFLC) **(right)** is plotted against CSF-serum ratio of albumin (Q Alb). Solid line indicates the upper reference value introduced in Figure 2. AI-CNS-D, other autoimmune disease of the central nervous system; CIDP, chronic inflammatory demyelinating polyneuropathy; CIS, clinically isolated syndrome; CNS-I, viral and bacterial CNS infection; GBS, Guillain-Barré syndrome; ME, metabolic encephalopathy; MS, multiple sclerosis; PP-PNP, paraproteinemic neuropathy and/or neuropathy with monoclonal gammopathy of unknown significance; P-CNS-I, post-infectious CSF syndrome.

In the 75 MS patients, Q KFLC was positive (i.e., above Q Alb-dependent reference value described in Figure 2) in 70 (93.3%) and Q LFLC in 53 (70.7%) (Figure 3). OCB were detected in 71 of 75 patients (94.7%) (Table 1) and IgG-index was elevated (>0.7) in 47 (62.7%) and intrathecal IgG synthesis according to Reiber was found in 48 (64%). One of the four OCB negative MS patients showed positive Q KFLC, and two of the five patients with normal Q KFLC showed positive OCB. Thus, either increased Q KFLC or CSF OCB or both were found in 72/75 (96%) of the patients with MS. Q KFLC was significantly more often elevated than the IgG index (*p* < 0.0001) and Q IgG according to Reiber (*p* < 0.0001).

Q KFLC was higher than the Q Alb-dependent upper reference value in 55.6% of patients with AI-CNS-D, in 80.0% of patients with CIS, in 23.1% of patients with CIDP, in 0% of patients with GBS, in 51.7% of patients with CNS-I, in 42.9% of patients with CNS tumor, in 36.8% patients with P-CNS-I, in 20.0% of patients with ME, in 21.4% of patients with PP-PNP, and in 0.9% of patients with NIND.

In comparison, Q LFLC was higher than the Q Alb-dependent upper reference value in 36.1% of patients with AI-CNS-D, in 40.0% of patients with CIS, in 7.7% of patients with CIDP, in 7.7% of patients with GBS, in 37.9% of patients with CNS-I, in 42.9% of patients with CNS tumor, in 21.1% patients with P-CNS-I, in 20.0% of patients with ME, in 7.1% of patients with PP-PNP, and in 0.9% of patients with NIND.

**Figure 4:**
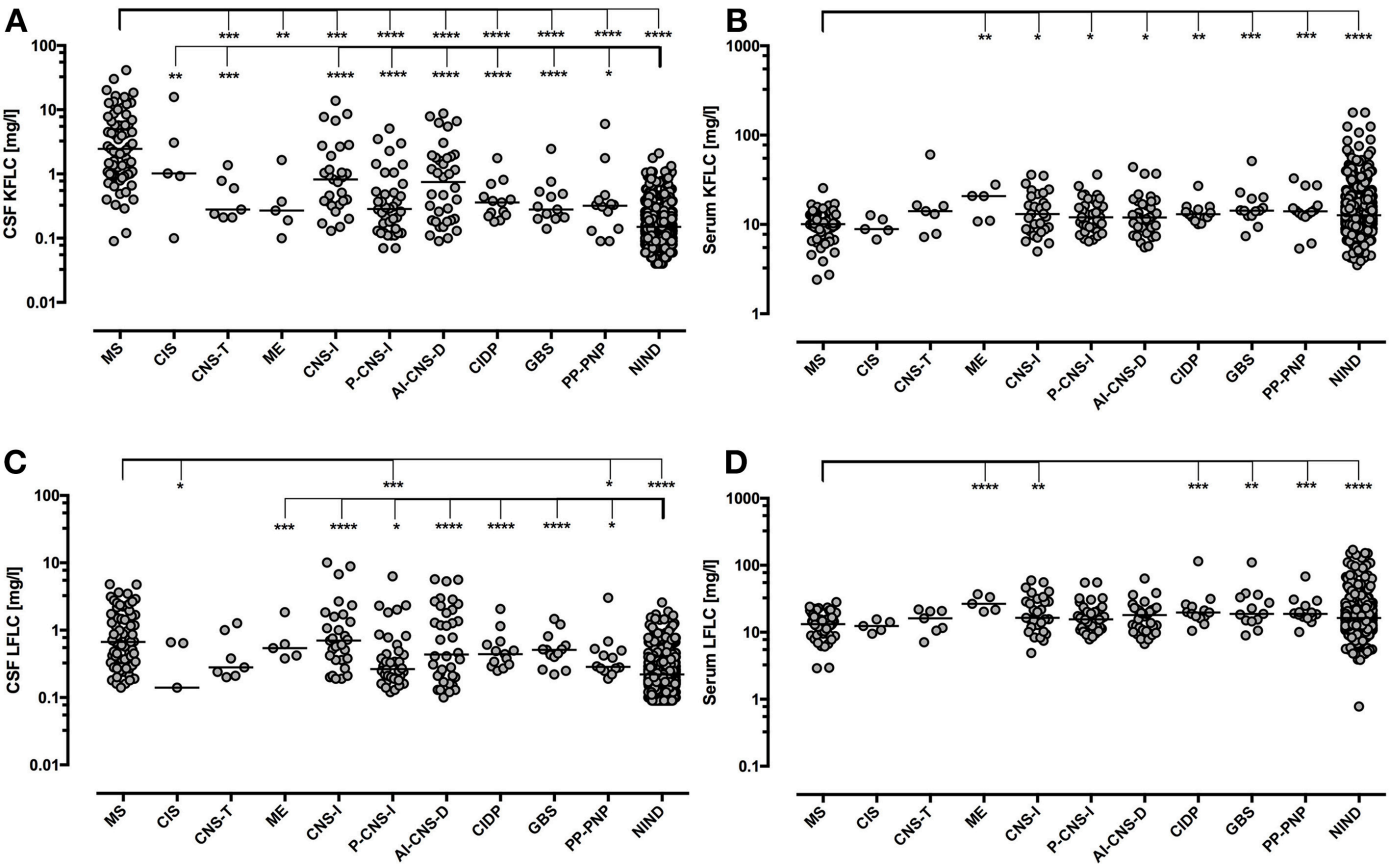
Immunoglobulin Free light chain levels in neurological diseases. Horizontal solid line indicates median. All groups have been compared to NIND and MS. Significant *P*-values for pairwise comparisons (Mann-Whitney U-test) are displayed. ^****^p < 0.0001, ^***^p < 0.001, ^**^p < 0.01, ^*^p < 0.05. AI-CNS-D, other autoimmune disease of the central nervous system; CIDP, chronic inflammatory demyelinating polyneuropathy; CIS, clinically isolated syndrome; CNS-I, viral and bacterial CNS infection; CSF, cerebrospinal fluid; GBS, Guillain-Barré syndrome; ME, metabolic encephalopathy; MS, multiple sclerosis; PP-PNP, paraproteinemic neuropathy and/or neuropathy with monoclonal gammopathy of unknown significance; NIND, non-inflammatory neurological diseases; P-CNS-I, post-infectious CSF syndrome. CSF **(A)** and serum **(B)** levels of KFLC as well as CSF **(C)** and serum **(D)** levels of LFLC are displayed.

Patients with MS showed significantly elevated CSF KFLC levels as compared with all other groups except for CIS and CSF LFLC levels were also elevated significantly as compared with NIND, PP-PNP, P-CNS-I, and CIS (Figure 4). CSF KFLC and CSF LFLC were also elevated significantly in other disease groups as compared with NIND. In contrast, serum KFLC and serum LFLC were significantly lower in cases of MS (*p* < 0.001) but showed otherwise no significant difference as compared with NIND.

### Diagnostic Accuracy of Different FLC Values, OCB, and IgG-Index for the Diagnosis of MS

**Figure 5:**
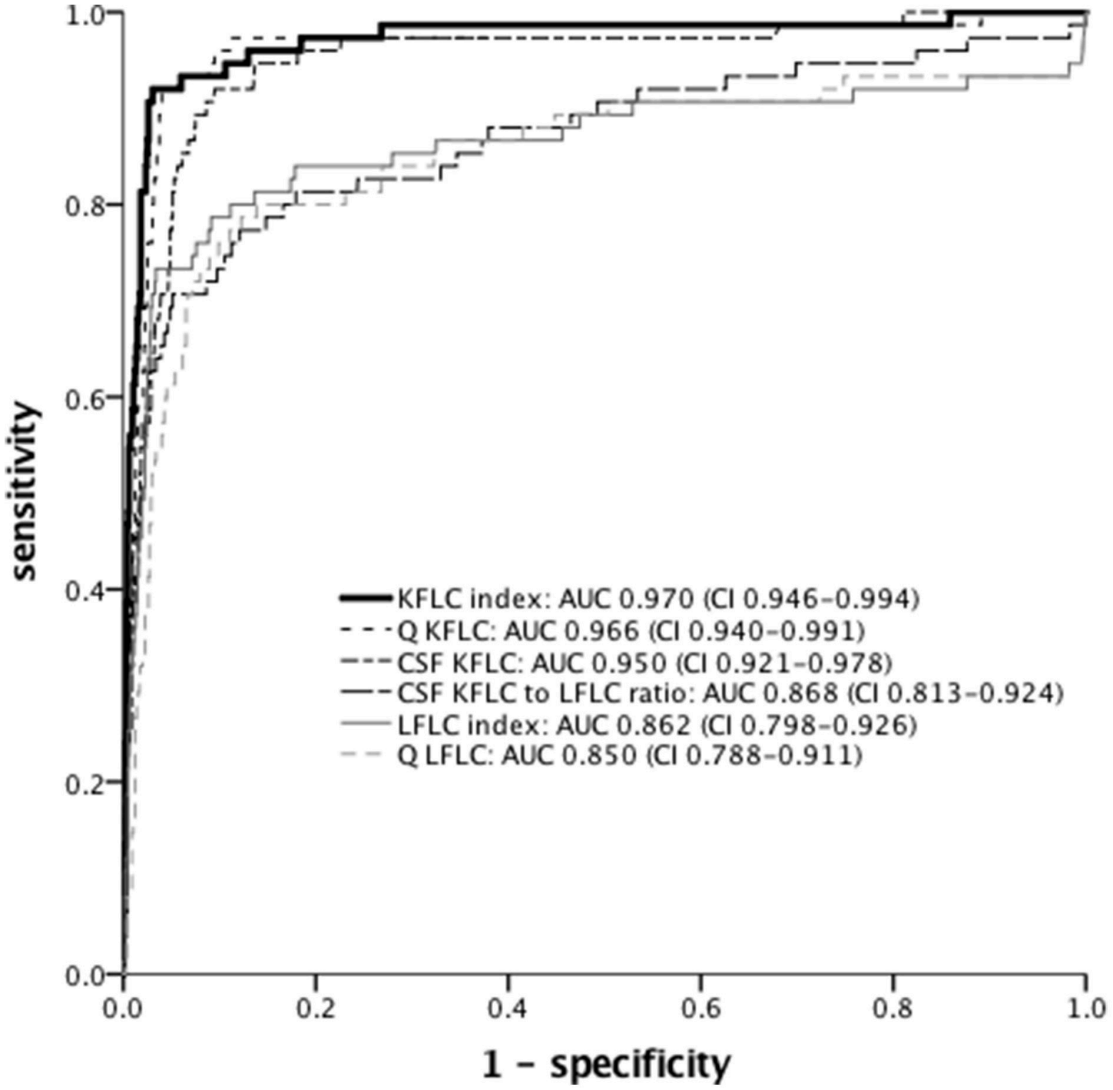
ROC curves of different FLC measures for discrimination between MS (*n* = 75) and non-MS (*n* = 1,149) patients. AUC and 95% confidence interval are given. KFLC, immunoglobulin kappa free light chain; LFLC, immunoglobulin lambda free light chain; Q Alb, CSF-serum ratio of albumin; Q KFLC, CSF-serum ratio of KFLC; Q LFLC, CSF-serum ratio of LFLC; KFLC index (Q KFLC/Q Alb); LFLC index (Q LFLC/Q Alb); ROC, receiver operating characteristics; AUC, area under the curve.

ROC analysis was performed to determine optimal cut-off levels of Q KFLC, Q LFLC, KFLC index, LFLC index, and CSF KFLC to LFLC ratio (Figure 5 and Table 2). To distinguish MS patients from all other patients, KFLC index (Q KFLC/Q Alb) showed the highest AUC with 97.0% (95% CI: 94.6–99.4) as compared to other quantitative FLC values. AUCs of KFLC measures were generally larger than those of LFLC measures.

**Table 2:** Sensitivity, specificity, positive, and negative predictive value with corresponding 95% confidence interval for different FLC values, IgG-index, and OCB regarding diagnosis of multiple sclerosis.

	**Sensitivity (%)**	**Specificity (%)**	**PPV (%)**	**NPV (%)**
Q KFLC > reference line^**^	93.3 (85.1–97.8)	93.7 (92.1–95.1)	49.3 (40.8–57.8)	99.5 (98.9–99.9)
Q LFLC > reference line^**^	70.7 (59.0–80.6)	95.7 (94.3–96.8)	51.5 (41.4–61.4)	98.0 (97.0–98.8)
Q KFLC > 60 × 10^−3^^*^	92.0 (83.4–97.0)	95.9 (94.6–96–9)	59.5 (50.0–68.5)	99.5 (98.8–99.8)
Q LFLC > 23.85 × 10^−3^^*^	78.7 (67.7–87.3)	87.7 (85.7–89.6)	29.5 (23.3–36.3)	98.4 (97.5–99.1)
CSF KFLC-LFLC ratio > 1.37* and/or CSF LFLC-KFLC ratio >12.07^*^	72.0 (60.4–81.8)	94.9 (93.4–96.1)	47.8 (38.3–57.4)	98.1 (97.1–98.8)
KFLC-index > 9.58^*^	92.0 (83.4–97.0)	97.0 (95.8–97.9)	66.4 (56.4–75.3)	99.5 (98.8–99.8)
LFLC-index > 5.85^*^	73.3 (61.9–82.9)	96.6 (95.4–97.6)	58.5 (47.9–68.6)	98.2 (97.3–98.9)
Q KFLC and/or Q LFLC > reference line^**^	94.7 (86.9–98.5)	92.7 (91.0–94.1)	45.8 (37.8–54.0)	99.6 (99.0–99.9)
OCB	94.7 (86.9–98.5)	93.3 (91.7–94.7)	48.0 (39.7–0.6)	99.6 (99.1–99.9)
IgG-index (>0.70)	62.7 (50.7–73.6)	98.3 (97.3–98.9)	70.2 (57.7–80.7)	97.6 (96.5–98.4)
Intrathecal IgG-Synthesis according to Reiber	64.0 (52.1–74.8)	98.8 (98.0–99.3)	77.4 (65.0–87.1)	97.7 (96.6–98.5)

*KFLC, immunoglobulin kappa free light chain; LFLC, immunoglobulin lambda free light chain; Q KFLC: CSF-serum ratio of KFLC; Q LFLC: CSF-serum ratio of LFLC, reference line: upper 99% quantile of the linear regression as described in Figure 2; IgG-Index: CSF-serum ratio of IgG (Q IgG)/CSF-serum ratio of albumin (Q Alb); KFLC-Index: Q KFLC/Q Alb, LFLC-Index: Q LFLC/Q Alb, OCB: cerebrospinal fluid oligoclonal bands of IgG class not detectable in serum*.

* value determined by ROC analysis,

** *introduced in Figure 2*.

Sensitivity, specificity, positive, and negative predictive values of all parameters investigated are given in Table 2. Of all markers investigated, OCB and Q KFLC (applying the Q Alb-dependent reference values described in Figures 2, 3) showed the highest sensitivities with 94.7 and 93.3%, respectively. Combination of Q KFLC and Q LFLC improved the sensitivity to 94.7%. IgG-index (above >0.7) and intrathecal IgG synthesis calculated according to Reiber showed the highest specificities of 98.3 and 98.8%, respectively. The KFLC index showed the highest combined sensitivity (92%) and specificity (97%).

## Discussion

Several findings support the role of B cells and intrathecal immunoglobulins in the pathogenesis of MS including (i) presence of B-cell infiltrates in the CNS parenchyma and meningeal tissues (41, 42), (ii) elevated CSF B-cell activation markers (e.g., the polyspecific intrathecal B cell response against neurotropic viruses MRZR as well as the B-cell attracting chemokine CXCL13) in MS (43, 44), (iii) upregulation of Ig-related genes in cortical sections of MS patients (45), and (iv) efficacy of B-cell targeting therapies (e.g., rituximab, ocrelizumab) (46, 47) as well as positive effect of apharesis treatments (plamapheresis, immunoadsorption) (48).

The presence of OCB in the CSF is known since the 1970s (49, 50) as the most prominent immunological hallmark of MS. In concordance with this, the CSF B-cell immunoglobulin transcriptome shows remarkable overlap with the corresponding immunoglobulin proteome (51–53) indicating immunoglobulin production by intrathecal B-cells. Besides intact immunoglobulins B-cells produce FLC as a by-product, which have been reported to be increased in the CSF of MS patients and proposed to be a quantitative diagnostic and prognostic biomarker for MS (17–26).

In line with previous studies from others and us (17, 18, 26) the present study confirms elevated intrathecal KFLC and LFLC production in patients with MS leading to high diagnostic accuracy. To allow an assessment of the utility and strengths of different FLC measures, we determined not only CSF and serum levels of FLC but also compared various FLC measures including CSF-serum ratio (Q KFLC, Q LFLC), FLC index (KFLC index, LFLC index), and CSF KFLC to LFLC ratio within one large cohort for their diagnostic accuracy in MS.

A significant correlation of CSF FLC levels (both KFLC and LFLC) with serum FLC levels and CSF-serum ratio of albumin (Q Alb) in NIND patients could be shown. Based on the large cohort of patients without any inflammatory CNS reaction we were able to introduce reference values of Q FLC (for both KFLC and LFLC) in relation to a wide range of Q Alb, which is a widely accepted quantitative measure of blood-CSF barrier function (54). Furthermore, ROC curve determined reference values were established for Q KFLC, Q LFLC, KFLC index, LFLC index, and CSF KFLC to LFLC ratio.

This study allowed to assess the diagnostic utility of the different FLC measures within one large cohort. Q KFLC (applying Q Alb- depending reference values) showed the highest sensitivity and the KFLC index showed the highest combined sensitivity and specificity. With regard to the diagnostic accuracy of Q KFLC in MS, however, applying the Q Alb-dependent reference values (introduced in Figure 2) showed no relevant difference as compared to the ROC curve determined cut-off value. This observation is possibly due to generally intact blood-CSF barrier function (normal Q Alb) in MS.

In general, KFLC showed higher diagnostic relevance in MS as compared with LFLC. This could be possibly explained by the dominance of KFLC in the human body, since the kappa chain is rearranged first during IgG production and is quantitatively more common. Furthermore, LFLC are usually dimeric in form while KFLC are generally monomeric but can exist as non-covalently linked dimer (55).

Here we compared not only the diagnostic accuracy of FLC values with OCB but also with other quantitative values of intrathecal IgG synthesis (IgG index and synthesis according to Reiber). Intrathecal KFLC and OCB showed nearly the same value (93.3 and 94.67%) with regard to diagnostic sensitivity in patients with MS, which is in line with earlier reports concerning diagnostic sensitivity of OCB in MS (56–58). As it is known for OCB (58), specificity of KFLC in MS diagnosis was significantly reduced when other inflammatory etiologies were considered.

Does the determination of FLC have any advantage over the established markers of intrathecal IgG synthesis? In comparison to OCB, measurement of KFLC is reliable, rapid, methodologically simple, can be performed using either ELISA or nephelometry, and can be applied in the clinical setting together with testing of basic CSF variables. In comparison to other quantitative parameters of intrathecal IgG synthesis (according to Reiber or IgG index), Q KFLC is more sensitive.

On the other hand, quantitative FLC values do not provide any insight into the clonality of intrathecal IgG, while the qualitative detection of IgG by immunoblotting can discriminate between monoclonal, oligoclonal, and polyclonal patterns.

In conclusion, CSF FLC may not replace OCB but may be supportive quantitative parameters in particular cases, for example in OCB negative MS cases or equivocal OCB findings.

Furthermore, this study again underlines the utility and accuracy of CSF examination in the diagnostic procedure of MS since very high diagnostic accuracy could be confirmed once again for well-established (OCB, IgG index) and demonstrated for promising (intrathecal KFLC) markers of intrathecal IgG production.

## Data Availability

The datasets generated for this study are available on request to the corresponding author.

## Ethics Statement

Written informed consent was obtained from all patients in accordance with the Declaration of Helsinki, and the study was approved by the ethics committee of the University of Ulm (No. 10/20).

## Author Contributions

MS, FM-Y, and HT were involved in the conception and design of the study. Data were acquired by FM-Y, MS, FB, JL, AL, MO, and HT. MS, FM-Y, UB, and HT were involved in the statistical methods and analysis. The first draft of the manuscript was designed by MS and HT, followed by critical revision of all authors. The final version for submission was approved by all authors.

### Conflict of Interest Statement

MS has received honoraria for speaking and/or travel from Bayer, Biogen, Sanofi Genzyme, and TEVA and research funding from the Hertha-Nathorff-Program and University of Ulm, none related to this study. JL has received honoraria for speaking and travel grants from Bayer, TEVA, CHDI, and the Movement Disorders Society. HT received funding for research projects, lectures, and travel from Bayer, Biogen, Genzyme, Fresenius, Merck, Mylan, Novartis, Roche, Siemens Health Diagnostics, Teva, and received research support from Hertie-Stiftung, DMSG, BMBF, University of Ulm and Landesstiftung BW. The remaining authors declare that the research was conducted in the absence of any commercial or financial relationships that could be construed as a potential conflict of interest.
